# The Rise of Syphilis Infections and Reinfections over a Decade (2009–2019) in the Bolognese Area: A Retrospective Analysis

**DOI:** 10.3390/microorganisms13020285

**Published:** 2025-01-27

**Authors:** Valeria Gaspari, Andrea Filippini, Gionathan Orioni, Martina Mussi, Miriam Anna Carpanese, Michelangelo La Placa, Bianca Maria Piraccini, Corrado Zengarini

**Affiliations:** 1 Dermatology Unit, IRCCS Azienda Ospedaliero-Universitaria di Bologna, 40138 Bologna, Italy; andrea.filippini2@unibo.it (A.F.); gionathan.orioni2@unibo.it (G.O.); martina.mussi2@unibo.it (M.M.); michelangelo.laplaca@unibo.it (M.L.P.); biancamaria.piraccini@unibo.it (B.M.P.); corrado.zengarini2@unibo.it (C.Z.); 2 Department of Medical and Surgical Sciences, University of Bologna, 40138 Bologna, Italy; 3 Dermatology Unit, AUSL Romagna, 48121 Ravenna, Italy; miriam.carpanese@ausl.romagna.it

**Keywords:** syphilis, treponema, surveillance, infection, reinfection, rates, epidemiology, PrEP

## Abstract

Syphilis has resurged globally, especially in urban areas of developed countries. This study analyses syphilis cases over a decade at an STD centre in Bologna, Italy, examining new diagnoses, reinfections, and impacts on high-risk subgroups, compared with national and European data. Data from 2009–2019 were retrospectively reviewed, including primary, secondary, early latent, late latent, and indeterminate syphilis cases, as per WHO guidelines. Cases of tertiary syphilis and serological-only diagnoses were excluded. Statistical analysis was conducted using IBM SPSS Statistics 26 with logistic regression and chi-square tests. A total of 1086 syphilis cases were identified, rising from 43 cases in 2009 to 157 in 2019—a 265% increase over the decade. In 2019, reinfections accounted for 23.7% of cases, primarily among men who have sex with men (MSM, 82.1%), with an HIV co-infection rate of 37.6%. The most affected age group was over 45 years. Bologna’s syphilis rates consistently exceeded European averages, with a higher median age, indicating unique transmission patterns and public health challenges. The high reinfection rate among MSM and older individuals emphasises the need for targeted public health initiatives. The sharp rise in cases highlights potential influences such as Bologna’s population dynamics and the increased use of Pre-Exposure Prophylaxis (PrEP). Focused public health efforts, particularly on high-risk groups, are critical to address this challenge effectively.

## 1. Introduction

Syphilis, a sexually transmitted disease (STD, also called sexually transmitted infection, STI) caused by the bacterium *Treponema pallidum*, has historically been a primary public health concern due to its severe complications and global prevalence [1,2,3,4]. After significant declines in incidence due to the advent of penicillin in the mid-20th century, syphilis has re-emerged as a public health threat, particularly in urban centres and among specific high-risk populations. This phenomenon has necessitated a renewed focus on the epidemiological patterns, risk factors, and effective management strategies for syphilis, especially in light of changing social behaviours and the complex interactions with co-infecting pathogens such as HIV [5,6].

This global resurgence observed over the past two decades is attributed to evolving sexual behaviours, decreased condom use, and the increasing use of HIV PrEP, which has significantly reduced HIV transmission but has been linked to risk compensation behaviours, potentially increasing vulnerability to other STIs, including syphilis [7,8,9].

High-risk groups, such as men who have sex with men (MSM) and individuals living with HIV, are disproportionately affected by this resurgence. MSM face heightened vulnerability due to biological factors, social network dynamics, and behavioural patterns that amplify syphilis transmission within this group [10,11]. HIV co-infection adds complexity, as it often presents with atypical clinical manifestations and increases the likelihood of reinfections [12,13].

Continuous monitoring and reports from national and international surveillance systems are fundamental for analysing trends and implementing changes and protocols to contain and mitigate the transmission of this pathology, along with other STDs. Specifically, Istituto Superiore di Sanità (ISS) serves as Italy’s national health institute and plays a pivotal role in monitoring STIs, including syphilis. Through its dedicated surveillance platform, the ISS collects data on STI cases from healthcare facilities nationwide, including hospitals, outpatient clinics, and specialised centres. This system ensures the consistent reporting of infections, categorisation by demographic and clinical characteristics, and integration with European-level databases managed by the European Centre for Disease Prevention and Control (ECDC) [14,15].

In this study, we examine syphilis trends and incidence in Bologna, Italy, an intriguing case study due to its diverse and transient population, including a significant university student community. In 2022, these groups accounted for over 15% of the total metropolitan population [16], contributing to the unique epidemiological landscape that may influence STI transmission dynamics differently than in more stable populations [17]. Additionally, Bologna’s robust healthcare infrastructure, with accessible public health services and a centralised STI centre, provides a comprehensive dataset for understanding the progression and management of syphilis over an extended period.

Moreover, the period analysed, 2009–2019, captures the dynamics of syphilis transmission in the pre-COVID-19 era, which saw an increase in high-risk behaviours. It also coincides with the advent of Pre-Exposure Prophylaxis (PrEP) in Italy, a potential factor influencing syphilis trends in certain populations. By conducting this decade-long analysis at a metropolitan STD centre, we also intend to compare local data with national and European statistics, focusing on patterns in syphilis prevalence, incidence, and reinfection with special attention given to high-risk groups, particularly MSM [8,12,18,19], who are known to be disproportionately affected by this STI. The analysis includes trends in new diagnoses, rates of reinfection, and co-infections, particularly with HIV, which complicates the clinical management and epidemiological tracking of syphilis.

An essential aspect of this study is the role of specialised centres in STI care. Unlike decentralised healthcare systems where STI care is dispersed across various providers, specialised centres like ours offer concentrated expertise, resources, and comprehensive datasets that enhance tracking, early detection, and intervention strategies for STIs. Centralised STI centres streamline patient care and contribute to epidemiological studies with more reliable data on transmission patterns, reinfection rates, and co-infections, particularly in high-prevalence settings. This study will highlight how specialised centres can play a pivotal role in identifying trends and implementing tailored interventions compared to decentralised systems, which may lack the focused resources necessary for effective STI management.

Understanding syphilis dynamics in Bologna is crucial not only for local public health officials but also for broader applications in similar urban settings. Insights from this study could improve targeted interventions, such as enhanced screening protocols, more effective educational campaigns, and optimised treatment strategies. As syphilis continues to challenge public health systems globally, studies like this one are essential for informing both local and international health policies aimed at controlling and eventually eradicating this re-emerging infection.

## 2. Materials and Methods

### 2.1. Study Design and Setting

This retrospective observational study was conducted at the STD centre in the Dermatological Unit of the IRCCS Azienda Ospedaliero-Universitaria of Bologna, Italy, a facility dedicated to the diagnosis, treatment, and management of sexually transmitted and venereological diseases. The centre serves a large urban population, including residents of the city and surrounding areas, as well as a significant number of university students and international visitors, which creates a dynamic and diverse patient population [16,20]. The study period spans from 1 January 2009 to 31 December 2019.

As part of the metropolitan clinical pathway for syphilis, the centre provides nearly all the STI care in the Bologna metro area, ensuring comprehensive tracking of cases within this population. The STD centre operates as both a direct-access facility and a reference centre, accepting patients referred by primary care providers and other specialised units. This dual role allows it to accommodate self-referred patients for screening and individuals referred for specialised care following initial assessments in primary or other healthcare settings.

For HIV management and prophylactic services, the centre collaborates closely with the Infectious Diseases Unit, which provides antiretroviral therapies and manages PrEP and post-exposure prophylaxis (PEP) programs. This collaboration ensures that patients receive holistic care for both STI and HIV-related needs within an integrated healthcare framework.

### 2.2. Data Collection

Data were systematically collected retrospectively from electronic and physical health records at the STD centre. All cases diagnosed with syphilis were included in the study according to the criteria defined by the World Health Organization (WHO) [21]. We analysed a range of variables to assess their association with syphilis reinfection. These variables were categorised into qualitative and quantitative types.

#### 2.2.1. Qualitative Variables

Gender: Male, female, undetermined.

Nationality: Italian, EU nationality, non-EU nationality.

Sexual Orientation: MSM (men who have sex with men), heterosexual.

Education Level: High (college degree), low, or university student.

Previous STIs: Yes, no (according to WHO classification).

HIV Status (regardless of the severity).

Type of Syphilis: Primary, secondary, early latent, late latent, indeterminate.

#### 2.2.2. Quantitative Variables

Age: Grouped into categories (0–14, 15–19, 20–24, 25–34, 35–44, over 45).

### 2.3. Diagnostic Criteria

The diagnosis of syphilis was confirmed through a combination of clinical assessment and serological tests. The primary diagnostic tools included the Treponema pallidum hemagglutination assay (TPHA), Treponema pallidum enzyme immunoassay (TP-EIA), and rapid plasma reagin (RPR), which are used to assess disease activity and monitor treatment response [3,22].

These tests were conducted according to the guidelines established by the Italian Ministry of Health and the European Centre for Disease Prevention and Control (ECDC) [23,24].

### 2.4. Case Selection Method

All patients who accessed our centre during the study period were included. Most of the study population came for screening and testing, often following sexual intercourse, the presence of venereological signs or symptoms [25,26,27], or referrals from other specialists for further investigation [28,29]. Accidental findings of syphilis during routine screenings were also included.

Cases of tertiary syphilis and those identified solely through positive serological tests without corresponding clinical symptoms were excluded. This exclusion applies to instances where a positive serology was observed but did not meet the diagnostic criteria for active syphilis based on the guidelines from national and European surveillance systems. Specifically, we excluded cases with isolated positive treponemal tests (e.g., TPHA or TP-EIA) that were not confirmed by non-treponemal reactivity (e.g., RPR) or lacked clinical evidence of disease activity [30,31,32]. We also excluded all cases with incomplete demographics.

Our focus was on cases with a clear clinical or serologically active infection, which includes primary, secondary, early latent, and late latent syphilis. Latent syphilis, though asymptomatic, was included if it met the criteria for confirmed infection (both treponemal and non-treponemal test positivity), aligning with standard definitions used in public health tracking. This approach ensured that we analysed cases with either clinical presentation or robust serological evidence of syphilis while avoiding overestimation from isolated serological reactivity that could indicate past, resolved infections or biological false positives.

Reinfected patients were classified as such if they had a documented previous infection in their electronic medical record or if an earlier diagnosis was confirmed in the laboratory followed by seronegativity and later re-conversion to seropositivity, thus supporting a new infection episode [33].

### 2.5. Data Analysis

Data were anonymised and analysed using IBM SPSS Statistics 26. The analysis included:

- Descriptive Statistics: To summarise the characteristics of the study population, including age, gender, and risk group distributions.
- Trend Analysis: To evaluate the trends in syphilis diagnoses over the study period.
- Univariate regression: A univariate analysis was performed to determine their association with the risk of reinfection.

### 2.6. Ethical Considerations

The study protocol was reviewed and approved by the local ethics committee of the University of Bologna under the protocol code InCOV-MTS-BO. All procedures performed in studies involving human participants followed the ethical standards of the institutional and/or national research committee and with the 1964 Helsinki Declaration and its later amendments or comparable ethical standards.

## 3. Results

The retrospective analysis revealed a growth in the epidemiology of syphilis from 2009 to 2019. A total of 1086 syphilis cases were recorded during this period. The data indicate an overall upward trend in syphilis diagnoses, notable variations in age and gender distributions, significant rates of reinfection, and high levels of HIV co-infection among diagnosed individuals, particularly in the MSM subgroup; however, it should be noted that the sexuality of the entire sample could not be determined: the definition of hetero/homosexuality was only possible in a percentage of patients. The category ’heterosexual men’, therefore, also includes male patients for whom it was not possible to ascertain sexual orientation. Global data are summarised in Table 1.

### 3.1. Epidemiological Trends

**Figure 1:**
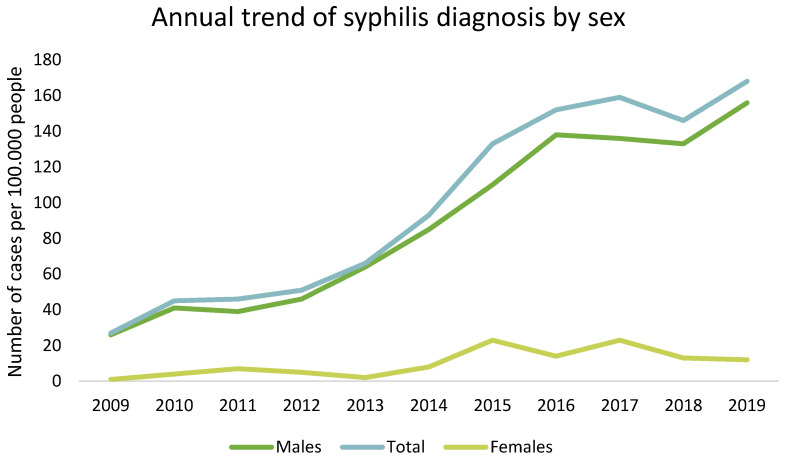
Trend of syphilis cases over the study period. This line graph illustrates the annual number of syphilis cases diagnosed per sex, highlighting the upward trend and identifying peak years of diagnosis.

Over the ten-year study period, the number of syphilis cases diagnosed annually increased progressively. In 2009, 78 cases were recorded, gradually rising to 156 by 2019. This represents a doubling of cases over the decade, with significant spikes observed in 2014 and 2019 (Figure 1).

### 3.2. Age and Gender Distribution

**Figure 2:**
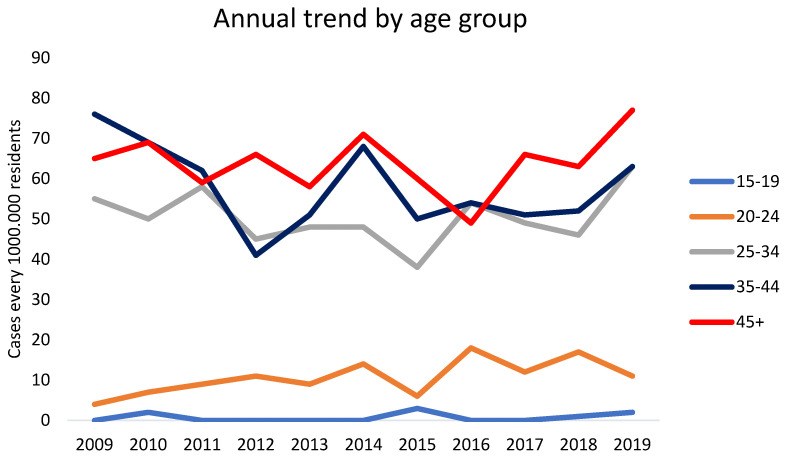
Line graph illustrating the yearly number of syphilis cases per age group.

**Figure 3:**
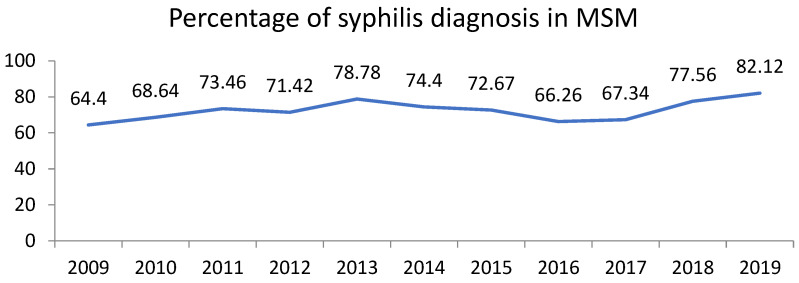
Line graph showing the percentage of syphilis cases in MSM among male cases.

**Figure 4:**
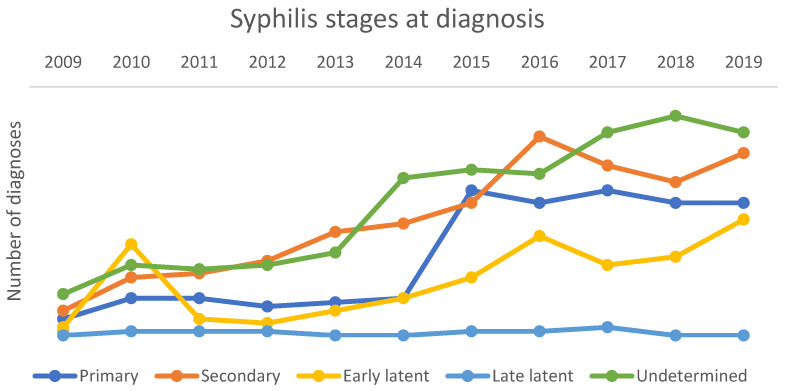
Trends in the stages of presentation at diagnosis for the entire population regardless of sex and sexuality, Bologna STD Centre, period 2009–2019.

The distribution of syphilis cases by age and gender highlighted specific at-risk populations. Most cases (82.9%) were diagnosed in males (Figure 1). The age group most affected was those between 25 and 50 years, which accounted for approximately 65% of all cases (Figure 2, Figure 3 and Figure 4).

### 3.3. Reinfection Rates

**Figure 5:**
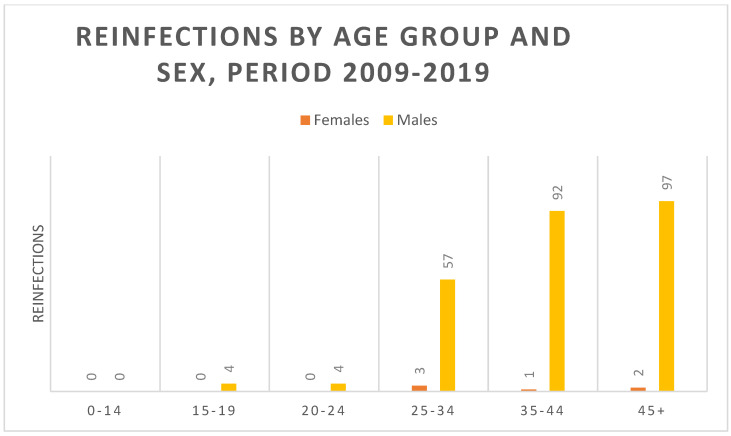
Reinfection rates among syphilis cases. Bar graph showing the percentage of reinfections among the total syphilis cases per year by age group and sex.

Reinfection rates accounted for 23.7% of all cases throughout the study period. Males represent the most significant category, with 250 cases making up 97.3% of the total. Specifically, MSM were disproportionately represented in reinfection statistics. Geographically, most reinfected patients (91%) were Italian. Considering males and females, the age group most subject to reinfection was the over-45s, representing over 38.5% of reinfected patients (Figure 5).

Our univariate regression analysis identified several variables related significantly to syphilis reinfection: HIV positivity (*p* < 0.0001), MSM category, previous STIs (*p* < 0.0001) and age over 35 (*p* < 0.001), particularly those in the 35–44 and over-45 age groups. Finally, a low instruction level was linked to reinfections (Table 2).

### 3.4. HIV Co-Infection

**Figure 6:**
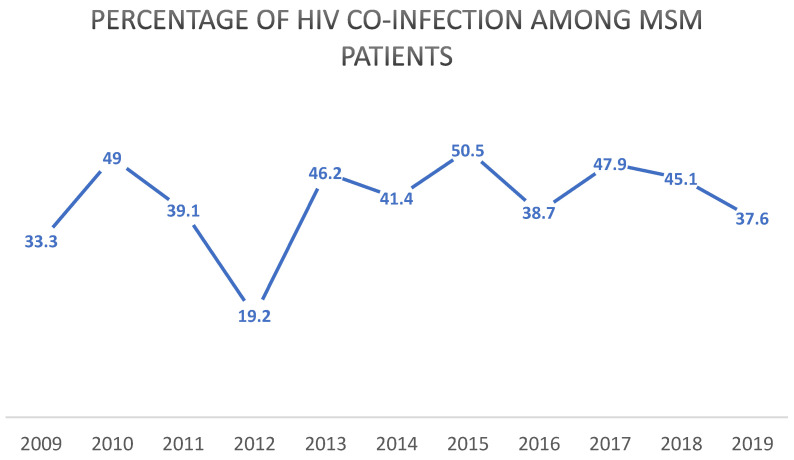
HIV co-infection rates among syphilis cases. Year-by-year line graph.

Co-infection with HIV was prevalent in the syphilis-diagnosed population, with an overall rate of 34.8%, which rises to 40.3% in the MSM subgroup (Figure 6).

### 3.5. Comparison with National and European Data

Comparative analysis with national and European data indicated that the syphilis incidence in Bologna was consistently higher than the averages reported elsewhere in Italy and across Europe.

## 4. Discussion

The increasing trend in syphilis diagnoses observed in the observed period reflects a broader resurgence in the Western world noted both across Europe and globally, as reported by the ECDC and other literature studies [34,35]. The analysis reveals some critical aspects, particularly the disproportionate impact on the MSM subgroup and the persistent challenges posed by high reinfection and HIV co-infection rates. These findings necessitate a nuanced examination of current public health strategies and their alignment with the observed epidemiological patterns.

### 4.1. Comparison with ECDC and Literature

The ECDC’s data highlight an overall increase in syphilis across many European countries, underscoring a shared public health concern that transcends national boundaries. The heightened prevalence in Bologna, which surpasses national [33] and broader European averages, suggests localised factors or intensified surveillance might influence these figures.

This trend could also reflect a more robust healthcare infrastructure and expertise, where centralised STI services and proactive screening by expert dermato-venereologist practices enhance case detection compared to decentralised systems in other regions, sometimes screened by blood samples [36,37,38]. Furthermore, the city’s demographic profile, including a sizeable transient university population and significant international tourism, may amplify exposure risks and reporting efficiency [34,39].

Literature on syphilis epidemiology consistently points to MSM as a particularly vulnerable group [35,40,41]. Studies highlight various factors contributing to this trend, including biological susceptibilities, network-level dynamics of sexual partnerships, and barriers to accessing effective prevention and treatment services. This disparity could reflect Bologna’s unique demographic profile, which includes a large transient student population and significant international interactions through tourism and business, which may contribute to higher STI transmission rates [28,29]. It underscores the need for enhanced interventions beyond initial treatment and addressing the underlying behaviours and circumstances facilitating repeated exposures.

### 4.2. HIV Co-Infection: A Dual Epidemic

The significant rate of HIV co-infection among syphilis cases, particularly among MSM, reflects a dual epidemic scenario that complicates clinical management and public health response [13,42,43]. This co-infection dynamic is well documented in the global literature, which describes the synergistic relationship between HIV and syphilis [44,45,46]. Both infections share common transmission routes and risk factors, enhancing the complexity of prevention and treatment strategies [47,48]. The findings from Bologna, which showed a 34.8% coinfections rate, slightly higher than the European average [49], echo the necessity reported in broader research for integrated screening and treatment approaches that address both conditions simultaneously, leveraging the overlap to maximise the public health impact.

### 4.3. Reinfection Rates and Public Health Implications

Our data, with a 23.7% reinfection rate, show that males constitute 97.3% of these cases, unlike the broader European trend, and show a higher reinfection rate in individuals over 35. This could be due to several factors that need tailored treatment and prevention initiatives for MSM and HIV-positive patients in this age group, which is probably lacking. Specific outreach and education campaigns for individuals over 35 to address prolonged risk behaviours and promote regular STI testing are needed.

It is challenging to trace the extent of reinfections, and the results in the literature are highly variable, ranging from 13.6% for the general population to 55.5% for some HIV-infected groups [33]. The substantial reinfection rates observed in Bologna suggest that current strategies may not sufficiently prevent subsequent infections. This observation aligns with the literature, which emphasises the importance of comprehensive health services, including behavioural interventions and consistent follow-up [33,50,51,52,53]. Reinfection rates also highlight potential gaps in patient education and engagement with health services, which are crucial for sustaining long-term health outcomes [50].

### 4.4. Implications for Public Health Strategy

The consistent rise in syphilis cases, particularly among high-risk groups, and the challenge of reinfections demand a re-evaluation of current public health strategies. Enhanced focus on tailored approaches that resonate with the needs and behaviours of at-risk populations, such as MSM, is critical. The integration of behavioural health interventions, community-based outreach programs, and stigma reduction strategies could address some of the social and cultural barriers that contribute to the syphilis epidemic.

Future strategies should also include digital health initiatives, such as mobile applications for STI testing reminders and virtual support groups, to engage younger and transient populations. Various authors have suggested that tech-driven interventions can significantly enhance STI prevention and monitoring in diverse communities [24,54,55,56].

### 4.5. Importance of Understanding Re-Infections

Recognising the characteristics of individuals prone to re-infections is crucial for developing targeted interventions. High-risk groups, such as MSM, require focused public health strategies, including regular screening, education, and prompt treatment to prevent transmission and re-infection.

### 4.6. Considerations of PrEP on Syphilis Epidemiology

Within the broader context of syphilis resurgence, the advent and increasing use of PrEP for HIV prevention warrants specific consideration. PrEP has been shown to significantly reduce the risk of HIV transmission in high-risk populations, particularly among MSM. However, its role in the epidemiology of other STIs, including syphilis, is complex. As suggested by some studies, the widespread use of PrEP may inadvertently contribute to increases in syphilis cases and other bacterial STIs [53,57,58,59]. This phenomenon could be due to behavioural changes such as the reduction in condom usage, known as risk compensation, where the perceived protection against HIV may lead to less cautious behaviours regarding other STIs in a specific high-risk group such as MSM [60,61]. Our data reflect this potential trend, with a sustained high incidence in the last years analysed concurrently with the advent of PrEP in Italy [59,62,63], and greater prevalence at the level of the MSM group, which uses it the most [64,65,66]. This pattern has been documented in other European cities and linked to similar sub-groups [56,57,58]. To mitigate these effects, PrEP programs must integrate regular syphilis screenings, risk-reduction counselling, and sustained condom promotion.

However, the implications are more profound, and it is impossible to explain this trend in such a simplistic way. However, this underscores the necessity for comprehensive sexual health strategies that address all aspects of STI prevention, particularly in populations using PrEP. Effective strategies might include enhanced STI screening and education programs that emphasise the importance of maintaining protective measures against a broad range of infections, not just HIV.

## 5. Conclusions

This monocentric, decade-long study of syphilis epidemiology provides data and insights into the dynamics of this infection within an urban European context. The significant findings—from the rising trend in syphilis cases to the stark prevalence of reinfections and the high rates of HIV co-infection, especially among MSM and older adults—highlight the complex and multifaceted challenges that syphilis continues to pose to public health and local communities.

PrEP use, while beneficial for HIV prevention, poses challenges for controlling infections like syphilis and could be associated with the rise in syphilis cases. Public health strategies must adapt to these changing behaviours, promoting regular STI screenings within PrEP programs and comprehensive safe sex practices.

Moreover, the insights gained call for robust epidemiological surveillance and further research into the underlying factors contributing to syphilis spread.

In conclusion, this study highlights rising trends in syphilis cases, the disproportionate impact on MSM and older adults, and the need for tailored public health strategies. These findings serve as a call to action for policymakers, practitioners, and researchers to reassess and reinforce their approaches to combating syphilis in the 21st century.

## Figures and Tables

**Table 1:** This table summarises the number of syphilis cases per year in Bologna from 2009 to 2019. It also includes the distribution of cases by gender, age group, HIV coinfection, and male sexual behaviours.

Year.	Total Syphilis Cases	Primary Syphilis	Secondary Syphilis	Early Latent Syphilis	Late Latent Syphilis	Indeterminate Syphilis	MSF	MSM	% Syphilis in MSM	% of HIV Among MSM
2009	43	4	6	2	0	10	16	6	64.4	33.3
2010	50	9	14	22	1	17	20	16	68.64	49.0
2011	60	9	15	4	1	16	31	12	73.46	39.1
2012	71	7	18	3	1	17	28	11	71.42	19.2
2013	84	8	25	6	0	20	29	17	78.78	46.2
2014	103	9	27	9	0	38	51	24	74.4	41.4
2015	116	35	32	14	1	40	78	32	72.67	50.5
2016	127	32	48	24	1	39	80	43	66.26	38.7
2017	132	35	41	17	2	49	85	51	67.34	47.9
2018	146	32	37	19	0	53	59	52	77.56	45.1
2019	157	32	44	28	0	49	79	56	82.12	37.6
Total	1086	214	311	148	7	368	556	320	72.46	40.73

**Table 2:** This table presents the results of a univariate regression analysis that examines the significance of various variables linked to syphilis reinfection.

Variables	Significance
HIV+	0.000
EU Nationality	0.300
Non-EU Nationality	0.999
Italian Nationality	0.999
Students	0.245
High Education Level	0.331
Low Education Level	0.031
MSM	0.001
Age 0–14	0.055
Age 15–19	1.000
Age 20–24	0.055
Age 25–34	0.999
Age 35–44	0.001
Age over 45	0.001
Previous STIs	0.000

## Data Availability

The data presented in this study are available on request from the corresponding author due to privacy reason.
